# 3D Printing for Mitral Valve Disease: A Systematic Review

**DOI:** 10.1093/icvts/ivag155

**Published:** 2026-05-26

**Authors:** Anastasiia Pavlykova-Chertovska, Shokoufeh Cheheili Sobbi, Vasyl Lazoryshynets, Peyman Sardari Nia

**Affiliations:** Department of Cardiothoracic Surgery, Cardiovascular Research Institute Maastricht (CARIM), Maastricht University, 6229 ER Maastricht, The Netherlands; Department of Cardiothoracic Surgery, Cardiovascular Research Institute Maastricht (CARIM), Maastricht University, 6229 ER Maastricht, The Netherlands; Amosov National Institute of Cardiovascular Surgery, National Academy of Medical Science of Ukraine, Kyiv 03038, Ukraine; Department of Cardiothoracic Surgery, Cardiovascular Research Institute Maastricht (CARIM), Maastricht University, 6229 ER Maastricht, The Netherlands

**Keywords:** 3D printing, mitral valve disease, surgical planning, cardiac imaging, valve modeling

## Abstract

**Objectives:**

Mitral valve disease remains one of the most prevalent and complex cardiac conditions, with treatment strategies varying based on anatomical and pathological factors. The heterogeneity in mitral valve morphology complicates standardized treatment, highlighting the importance of procedural planning and surgical training. This systematic review evaluates current techniques, materials, and clinical applications of 3D printing in mitral valve disease, with a focus on procedural planning and simulation.

**Methods:**

A comprehensive literature search of PubMed and MEDLINE databases was conducted to identify studies published from 1996 to August 2025. A total of 63 studies were included, all involving 3D printing technologies related to mitral valve disease. Extracted data covered imaging modalities, software tools, printing techniques, materials used, total time required for model creation, and clinical applications.

**Results:**

CT, 3D transoesophageal echocardiography, and MRI were the main imaging modalities, with CT being the most common. Data segmentation and model generation were performed using software such as Mimics, 3D Slicer, and Philips QLAB. The primary 3D printing techniques were stereolithography, fused deposition modelling, and PolyJet, using photopolymer resins and thermoplastics. Total processing time—from image acquisition to model completion—ranged from 45 min to 72 h, depending on complexity. Applications included procedural planning, surgical training, and the development of medical and simulation-based educational devices.

**Conclusions:**

3D printing enhances mitral valve surgery by enabling precise planning and training. Although challenges like standardization and accessibility persist, advances in imaging, software, and materials are expected to expand its clinical impact.

## Graphical abstract

**Figure:**
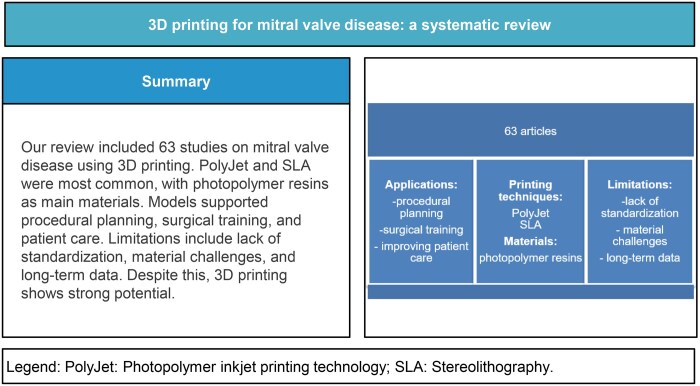

## INTRODUCTION

Mitral valve disease is the most common valvular pathology [1]. The diseases of the mitral valve are heterogeneous and therefore, different intervention modalities exist. The most common pathology of the mitral valve is mitral valve regurgitation (MR) [2]. Mitral valve regurgitation is commonly divided into primary (degenerative) and secondary (functional) [3]. For primary MR, it has been established that mitral valve repair is superior to replacement [4]. In the past decades, different surgical and transcatheter techniques have been developed [5]. However, the complex anatomy of the mitral valve, heterogeneity of anatomic lesions, and variety of repair techniques have hindered the standardization of interventions. This is reflected by a strong association between volume and outcome across mitral valve repair interventions, whether surgical or transcatheter [6].

Recent translational advances in 3D-imaging reconstruction, 3D printing, and simulations have made it possible to apply these technologies for procedural planning, simulation, and training of surgeons [7]. This could make it possible to standardize training, but also the intervention of MR.

The aim of this systematic review is to provide an overview of techniques, development, and application of 3D-printing (additive manufacturing) in mitral valve disease for procedural planning and simulation.

## METHODS

This systematic review adheres to the PRISMA guidelines [8]. It was registered in the PROSPERO database to promote transparency. Registration number is CRD420251155709.

**Figure 1.**
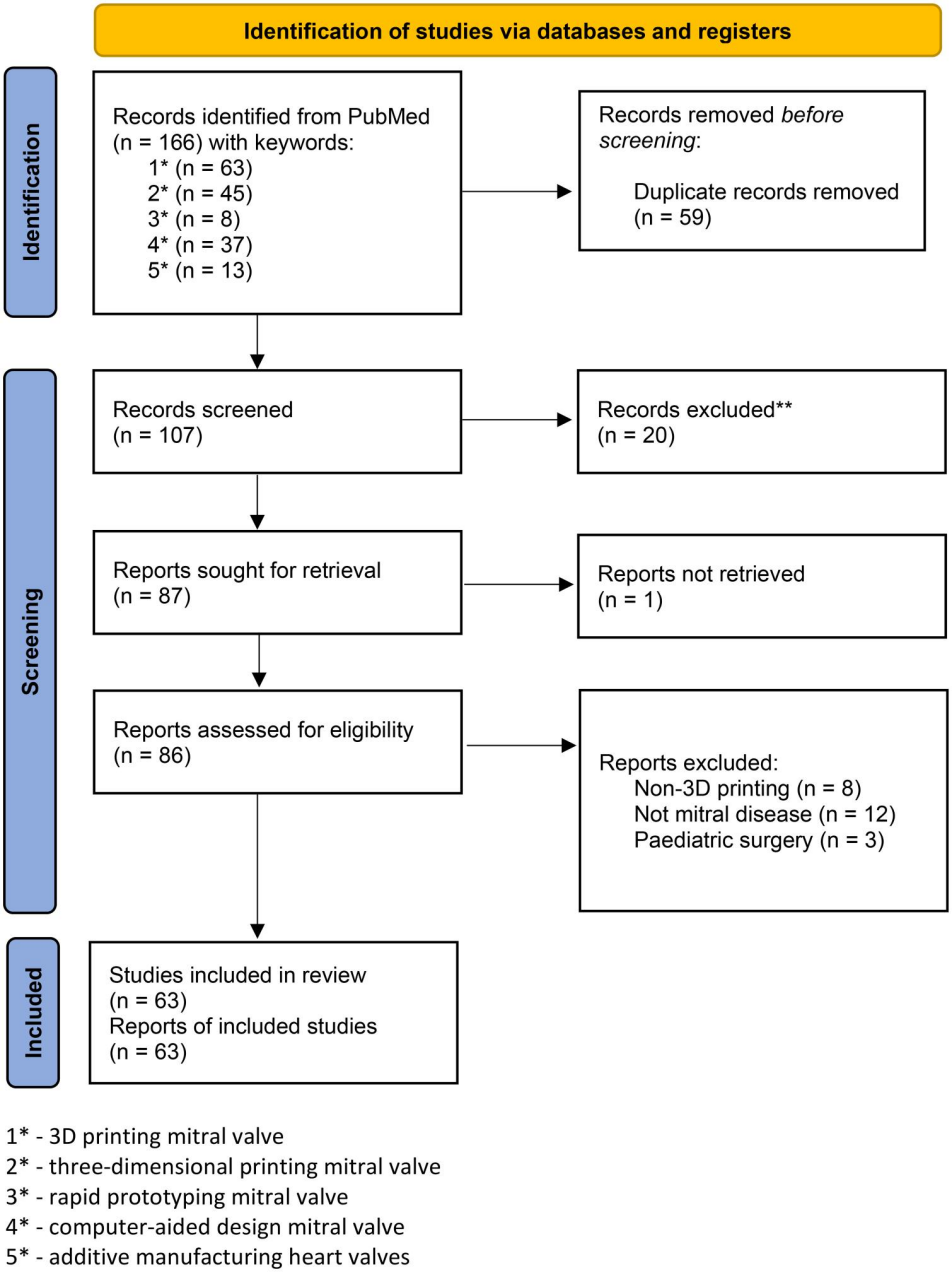
PRISMA Flow Diagram

The search was independently conducted by two authors (Pavlykova-Chertovska A. and Cheheilli Sobbi S.) using the PubMed and MEDLINE databases up to the end of August 2025. The search strategy incorporated both Medical Subject Headings (MeSH) and relevant keywords. Additionally, the reference lists of retrieved articles were screened to identify further eligible studies (Figure 1).

### Including criteria

We performed an overview of all available studies from 1996 to the end of August 2025. Inclusion criteria were full-text papers in the English language. “3D printing mitral valve”, “three dimensional printing mitral valve”, “rapid prototyping mitral valve”, “computer-aided design mitral valve”, “additive manufacturing heart valves”—keywords were used in the search.

### Excluding criteria

Our excluding criteria were all systematic and non-systematic reviews, non-full text publications and papers, which were not about mitral valve disease or not in English language.

### Data acquisition

Following the analysis of all articles based on keywords, a comprehensive database was created, capturing information such as the first author, publication year, and article type. The research purposes and results were also included. Special focus was placed on the techniques of 3D printing, materials, methods used for obtaining 3D images, software, and the time required for printing. Data extraction was performed independently by two authors (Pavlykova-Chertovska A. and Cheheilli Sobi S.) using Microsoft Excel. All records were imported into Zotero software for reference management. Title and abstract screening, followed by full-text assessment, were conducted independently by the same two reviewers.

### Quality assessment

Risk of bias was assessed using a mixed-methods approach. For non-comparative feasibility studies, methodological quality was evaluated qualitatively. For comparative non-randomized studies, the Newcastle-Ottawa Scale (NOS) was applied. Discrepancies were resolved through discussion and consensus, with continuous oversight and consultation provided by the third author (Sardari Nia P.) throughout the review process.

## RESULTS

The earliest research on 3D printing in cardiovascular pathology dates back to the 2000s [9]. However, the first mention of stereolithography of mitral valves was published in 1996 [10]. At that time, stereolithography and liquid polyacrylic polymers were the most commonly used techniques for creating the first 3D-printed heart models. Since then, technology has been constantly evolving. Each year, the variety of software, printing techniques, models, and materials continues to expand.

**Figure 2.**
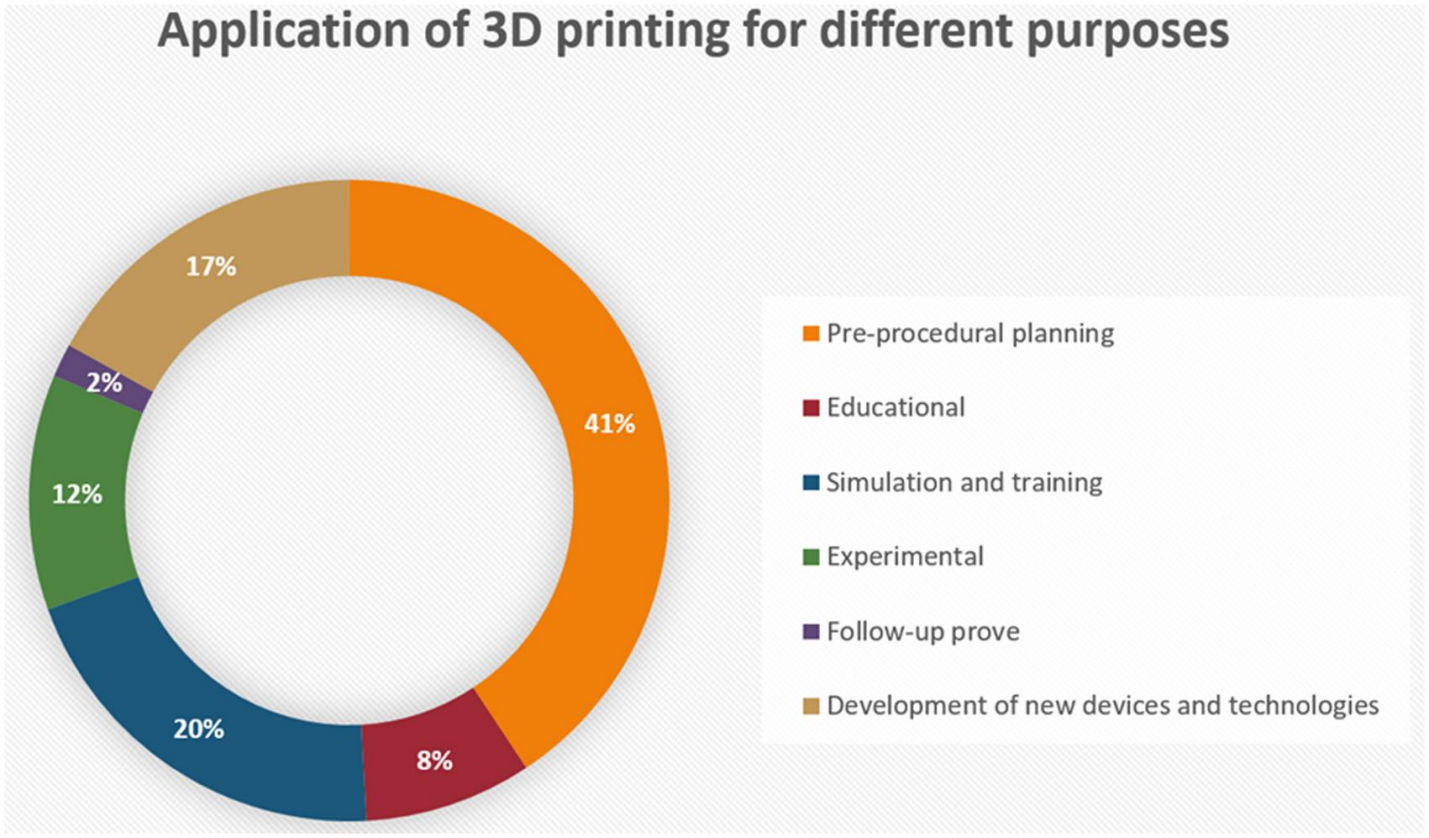
Different Purposes of 3D Printing Application

The included articles were categorized according to their primary purpose as follows: in 26 (41%) cases, 3D printing was used for preprocedural planning; in 13 (20%) cases, for simulation and training; and in 11 (17%) cases, for the development and testing of new devices and technologies. Similar distributions were observed for experimental research and educational purposes, accounting for 8 (12%) and 5 (8%) cases, respectively. The least frequently reported application was the use of 3D printing for postoperative follow-up evaluation. All results are summarized in **Figure 2**.

With regard to clinical applications, several important findings can be highlighted.

**Figure 3.**
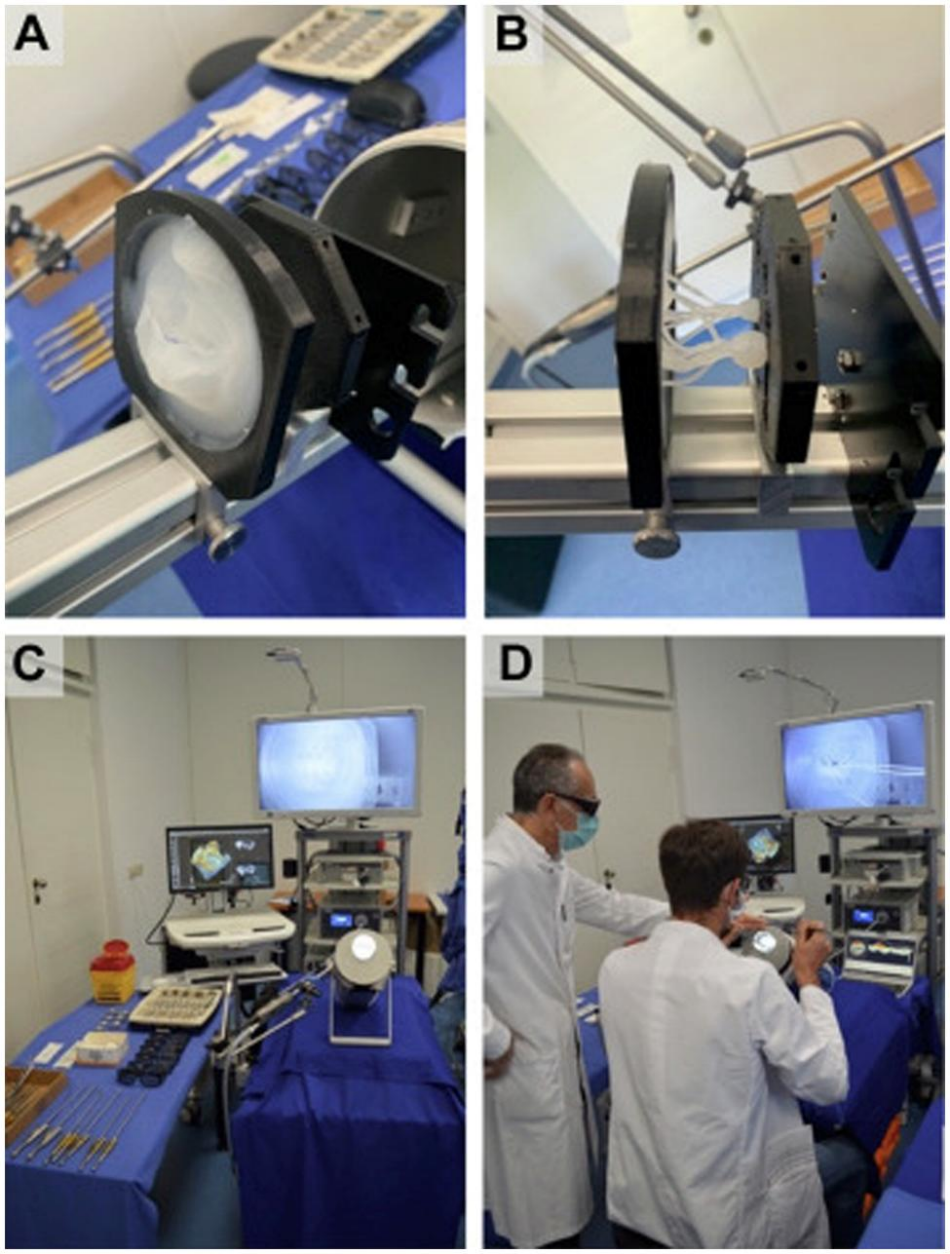
Simulation Setup. (A, B) Placement of the Valve Replica in a 3-Dimensional (3D) Printed Frame into the Simulator and Fixation of the Papillary Muscles. (C) The Complete Simulation Setup with Surgical Instruments, 3D Transoesophageal Echocardiography Examination Data, and 3D Endoscopic View on the Silicone Valve, Which is Mounted in the Simulator. (D) A Mitral Valve Expert is Able to Reconstruct the Valve Model While Interacting with a Colleague. Samantha Fisher et al

Samantha Fisher et al [11] reported that training on a patient-specific printed mitral valve model shortened intraoperative valve assessment by at least 1 min (**Figure 3**).

Similarly, 6 other publications described a reduction in intraoperative time, a lower risk of complications, and overall improved surgical outcomes due to preoperative selection of surgical technique, device choice, and hands-on training with individualized models.

Angel Babu et al [12] emphasized that 3D printing increases operator confidence during interventions. In addition, several studies confirmed that 3D printing enabled accurate diagnosis in cases where conventional imaging methods were inconclusive. Five publications directly compared the accuracy of printed models with *in vivo* findings, both intraoperatively and via 3 D TEE, demonstrating a high degree of correlation. Two studies reported particularly successful integration of 3D-printed models with the MitraClip device, allowing preprocedural testing that contributed to positive outcomes.

Educational applications were also frequently emphasized.

**Figure 4.**
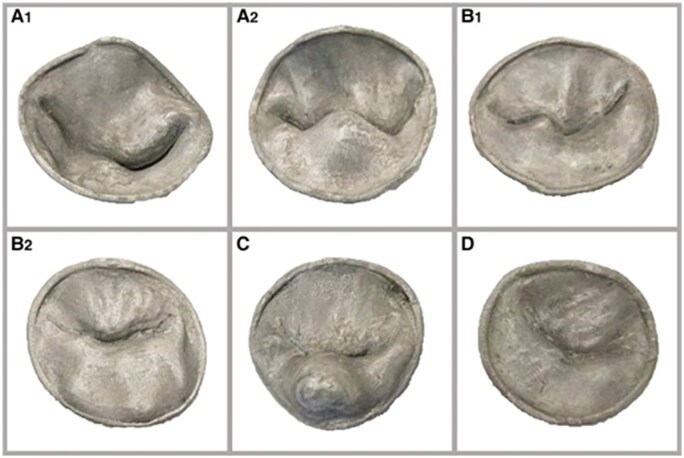
3D Printed Models of Mitral Valve. Ziad Bulbul et al

Ziad Bulbul et al [13] demonstrated that 3 D-printed models significantly improved training quality and anatomical understanding among junior physicians and fellows (**Figure 4**).

The initiation of 3 D printing process generally begins with acquiring high-quality imaging data.

**Figure 5.**
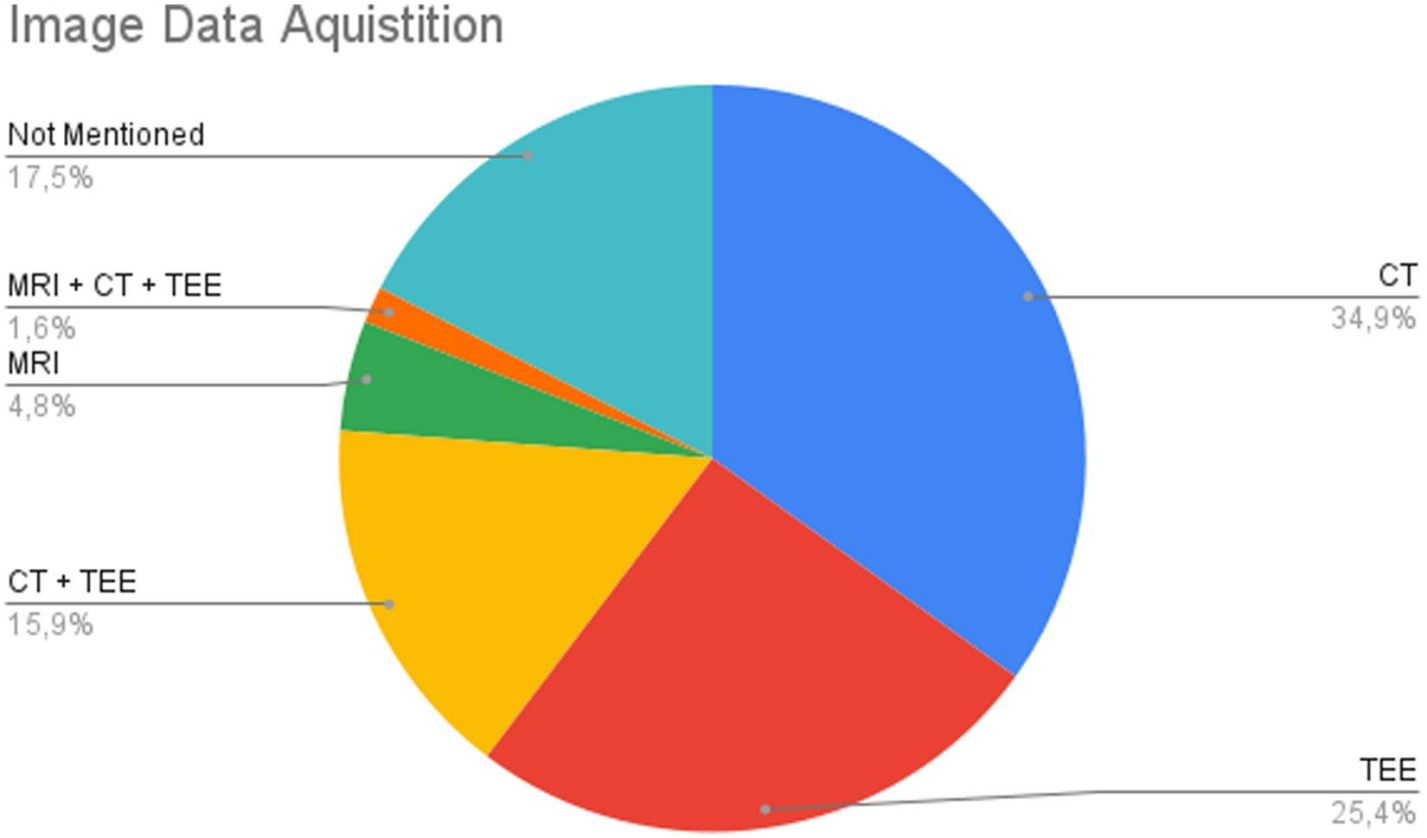
Frequency of Imaging Methods Used in Analyzed Studies

In our analysis, CT imaging was the most commonly used modality for 3D printing of mitral valves, reported in 22 studies. While CT remains the dominant approach, 3D transoesophageal echocardiography (3D TEE) is rapidly emerging as an alternative, appearing in 16 studies. Both CT and 3D TEE were used together in 10 reports. Magnetic resonance imaging (MRI) was less commonly employed, primarily as a supplementary method, and was reported in 3 studies. All 3 modalities (CT, TEE, and MRI) were used together in a single study. Notably, 11 publications did not specify the imaging method used to obtain patient data. The distribution of imaging modalities is shown in **Figure 5**.

After obtaining high-quality images, the next step is segmentation and conversion into the STL format. As most images are initially stored in DICOM format, various software tools are required for this process. The choice of software depends largely on the imaging modality.

For 3D printing of mitral valve models, the following tools are most commonly used:

- Philips QLAB—frequently employed for ultrasound data, including TEE. It enables segmentation and conversion of images into 3D models for printing.
- Mimics and 3D Slicer–widely used for CT and MRI imaging, providing comprehensive functionality for segmentation, visualization, and model generation.
- SolidWorks and Vesalius 3D—additional software solutions used for model processing.

Once converted into STL format and supplemented with support structures, the printing process can commence. Various 3D printing techniques are utilized, each selected based on the specific requirements and intended application. With a vast number of techniques currently available, let us focus on those most commonly utilized in the additive manufacturing of mitral valve disease.

SLA, or stereolithography the most widely used 3D printing method, which is based on the polymerization of liquid resin using light.

Two common modifications of SLA are fused deposition modelling (FDM) and digital light processing (DLP).

In FDM, a thermoplastic filament is heated and extruded layer by layer to build the model. FDM is typically used for creating functional prototypes or less intricate models compared to SLA. Although it’s not as precise, FDM is still useful for producing patient-specific models of the heart and blood vessels, as well as for creating tools or supports used in surgery.

On the other hand, DLP uses a digital projector to cure liquid resin in a layer-by-layer fashion. DLP is known for its speed and high precision, making it ideal for creating highly detailed cardiac models. Like SLA, DLP is often used to create patient-specific models for surgical planning, including heart valves, chambers, and vasculature, enabling surgeons to assess and plan complex operations in advance.

Powder-bed fusion 3D printing technologies that employ a laser to create parts layer by layer are known as selective laser sintering (SLS) and selective laser melting (SLM). The main difference between these two methods lies in the melting temperature and the materials used.

The principle of SLS is that the laser heats the powdered material just below its melting point, causing the particles to fuse together, or “sinter”, and form a solid part. This process creates a strong bond between the particles, but the material does not fully melt. The materials used in SLS include thermoplastics (such as nylon, polystyrene, and TPU), certain metals, and composite materials. These materials are typically in powdered form, which allows them to be easily sintered.

In contrast, in SLM, the laser completely melts the powdered material, causing the particles to fuse into a fully dense solid structure. This process results in a part that is entirely melted and then solidified, leading to higher density and enhanced mechanical properties. Accordingly, the materials used in SLM are various types of metals, such as stainless steel, titanium, aluminium, and other metal alloys.

Regarding drop-on-demand deposition methods, *InkJet* and *PolyJet* are the primary techniques. The key distinction between these methods lies in the type of material utilized, the deposition process, and the resultant characteristics of the 3D-printed components.

In a typical *InkJet* 3D printer, material is ejected in droplets from print heads onto the build platform. Depending on the ink type, these droplets can be cured or hardened using ultraviolet (UV) light. Inkjet technology is limited to printing a single material at a time, which makes it suitable mainly for single-material applications. It typically uses liquid inks or resins, with the resolution and surface quality of the printed object being influenced by the material’s properties. This method is commonly used for creating basic prototypes, models, or low-detail visual representations.

As for *PolyJet*, this technology, similar to inkjet printing, uses print heads to deposit material, but instead of conventional ink or liquid, it jets layers of photopolymer resin. After each layer is deposited, UV light is used for instant curing. PolyJet offers much higher resolution and finer detail, known for its ability to produce highly detailed, smooth surfaces with micrometer-level precision. A key advantage of PolyJet is its capacity to use multiple materials simultaneously within a single print job, enabling the creation of parts with diverse material properties. These parts can range from rigid to flexible, transparent to opaque, and even mimic rubber-like textures. PolyJet is particularly suited for applications that require detailed, multi-material components, such as medical models or complex product prototypes.

In our analysis, the most commonly employed 3D printing techniques were PolyJet and SLA, reported in 17 and 10 studies, respectively. The FDM method appeared in 5 articles, while FDM combined with silicone casting was reported in 4 studies. DLP and SLS were referenced in 2 and 3 studies, respectively. SLM and InkJet were rare, each appearing in only one study. In 19 articles, the specific printing method was not specified.

**Figure 6.**
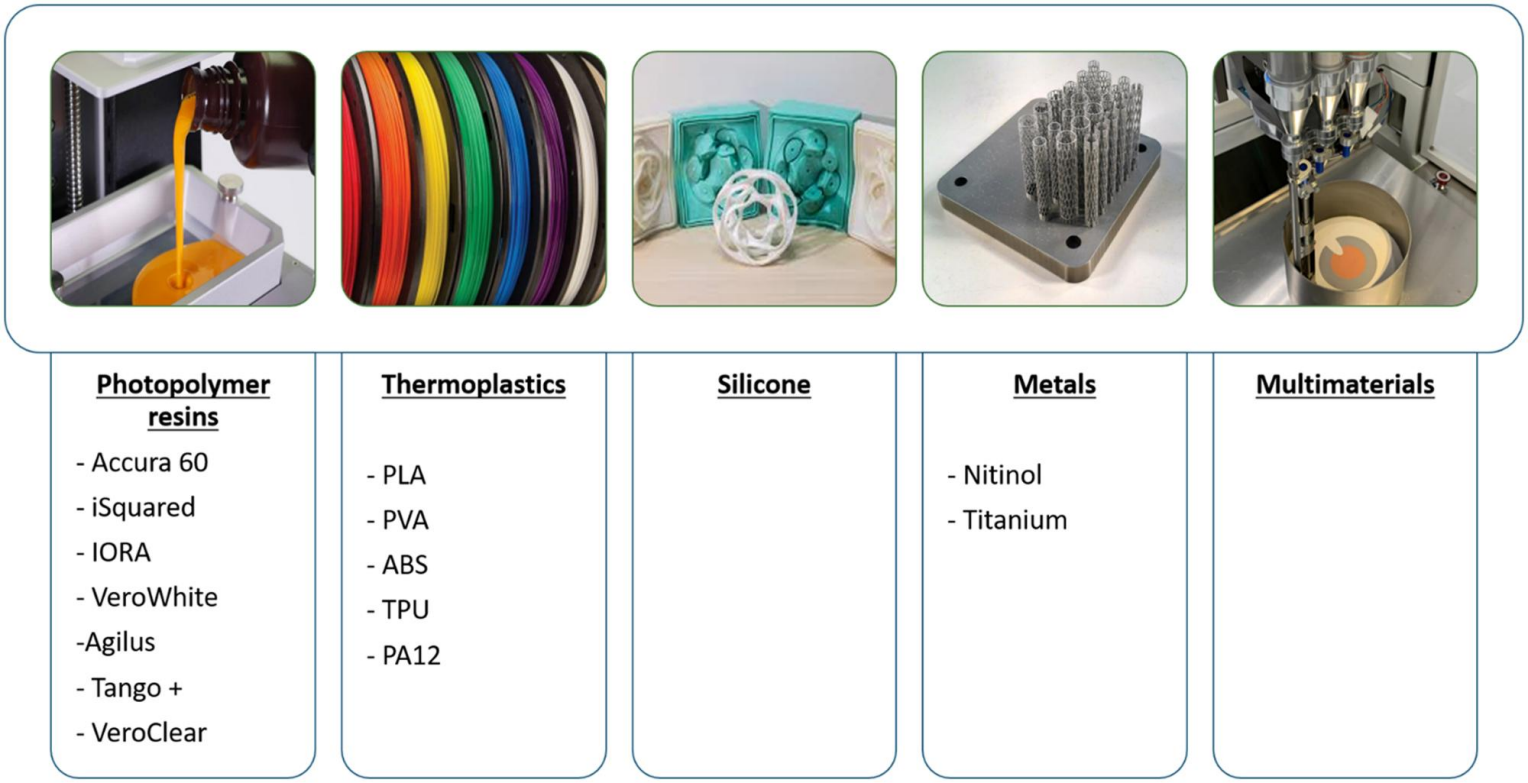
Different Types of 3D Printing Materials

The choice of material for 3D printing is closely related to the selected technique and intended application. To facilitate clarity, these data are presented in Figure 6.

According to this table, the most commonly used materials were photopolymer resins (reported in 18 articles) and thermoplastics (15 articles). Metals and silicones were used less frequently, in 2 and 4 articles, respectively. Multi-material combinations (e.g., silicone with resin, or metal with silicone) were described in 4 articles.

Following printing, the models underwent post-processing, which may include:

- Removal of supports (for SLA or FDM prints).
- Cleaning, particularly for photopolymer models.
- Post-curing under UV light to enhance strength.
- Sanding and painting to improve appearance and texture.

**Figure 7.**
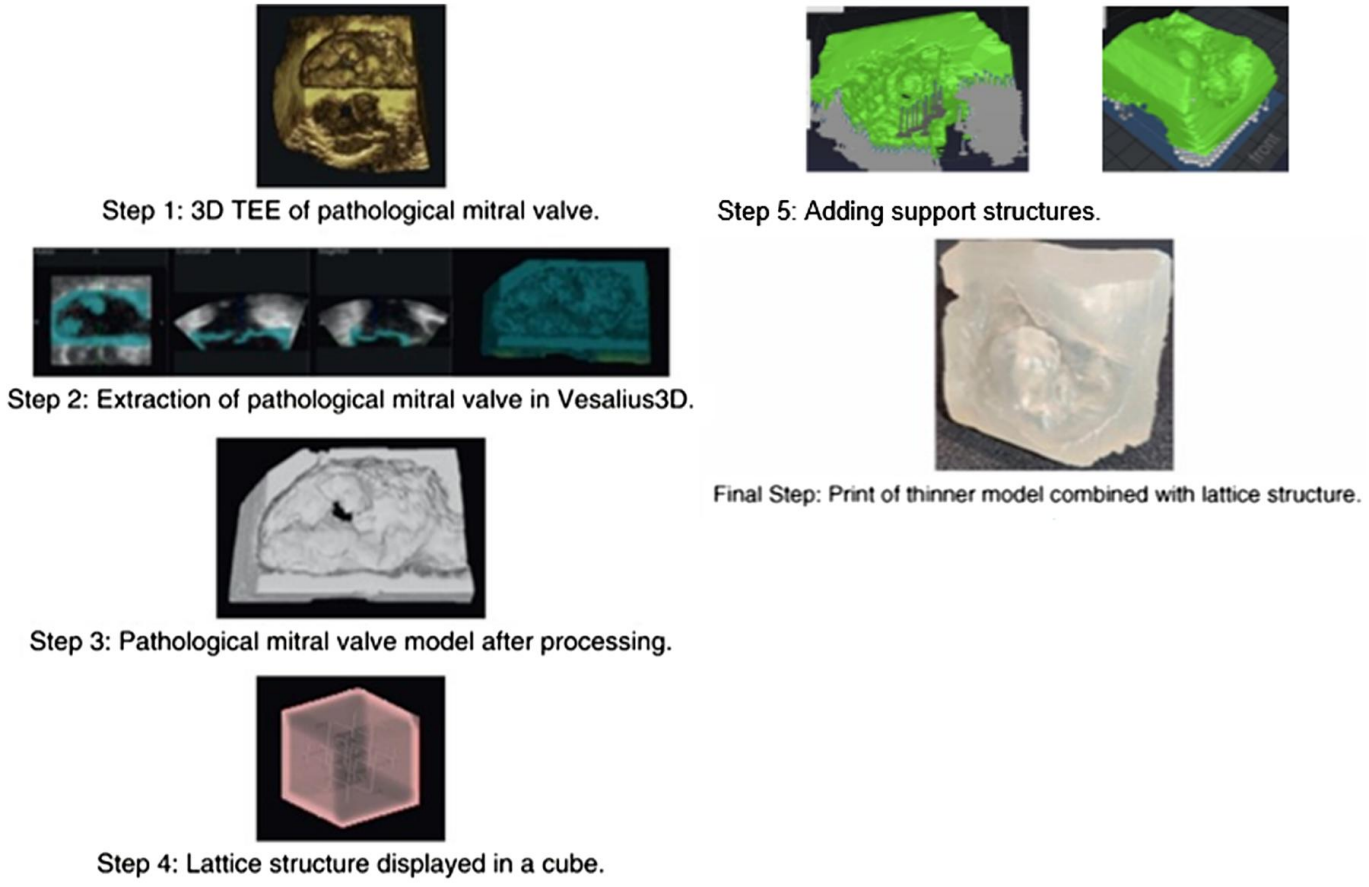
Direct 3D Printing Process of Soft Patient-Specific Mitral Valve

Shokoufeh et al [14] present a comprehensive three-dimensional direct-printing methodology for the fabrication of a mitral valve in soft material, with all processing steps detailed in **Figure 7**.

**Figure 8.**
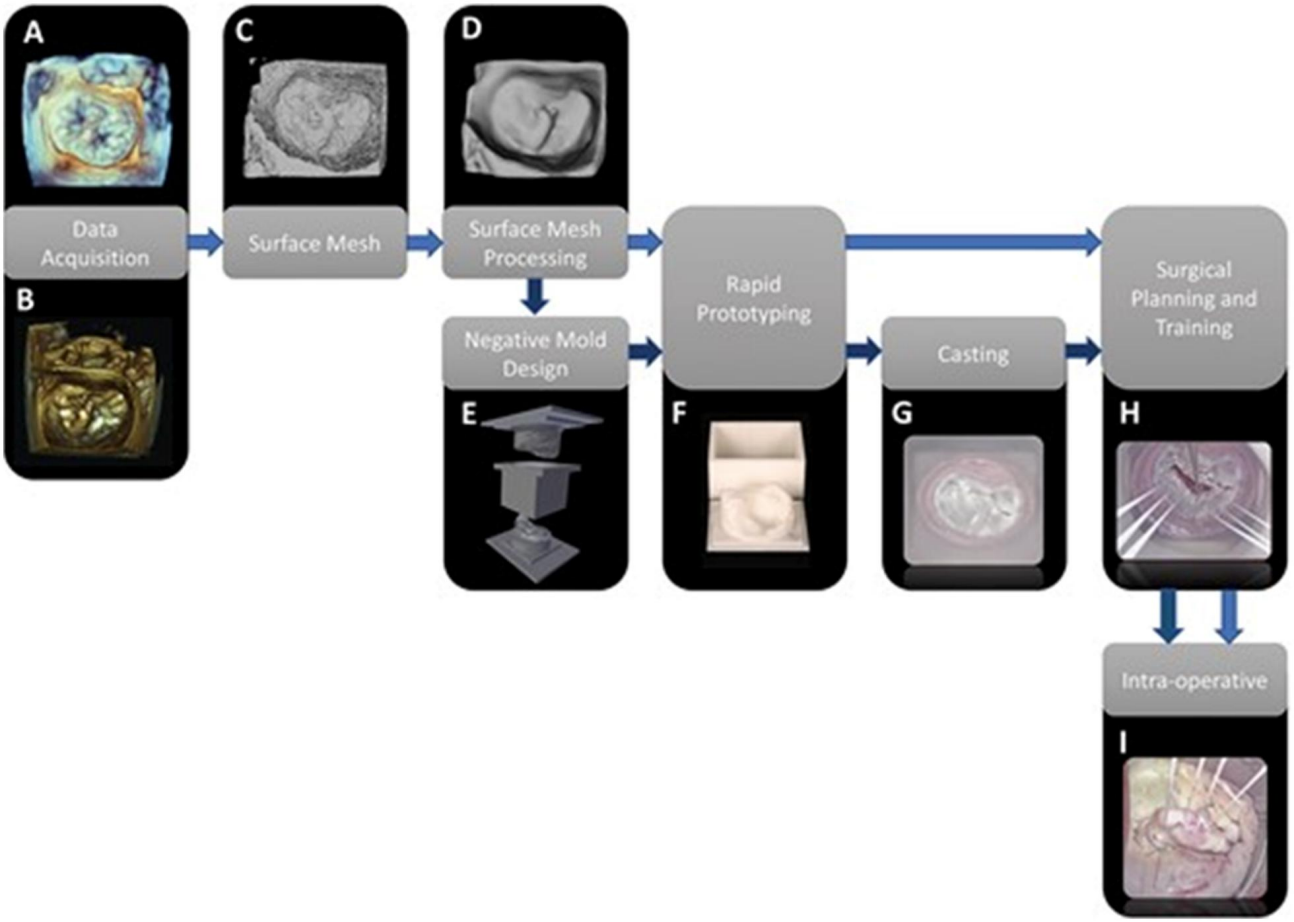
Overview of the Developed Process, Showing the Subsequent Steps to Create Pliable and Non-Pliable Mitral Valve Replications. Sardari Nia et al

Sardari Nia et al visually present a viable alternative fabrication approach based on negative molding followed by silicone casting, with the entire workflow illustrated in **Figure 8**. The study also provides a clear step-by-step overview of the negative moulding technique, from medical image acquisition and digital model generation to mould fabrication and production of the final silicone valve [1].

Time and cost are additional critical factors to consider. The duration of model fabrication depends on both the printing method and the material used. Inherently, 3D printing remains a time-consuming process. One of the objectives of this review was to identify the fastest, most accurate, and most cost-effective approaches. Based on our analysis, the average time required was less than 12 h across methods and materials. The fastest technique was PolyJet with multimaterials, which produced a model in only 45 min using silicone and embedded umbilical cells. In contrast, the longest duration was reported for selective laser melting (SLM) with titanium, requiring up to 72 h.

With respect to cost-effectiveness, none of the included studies reported detailed data on the financial costs associated with 3D printing.

The majority of included studies were feasibility or technical investigations and showed a high risk of bias due to small sample sizes and the absence of control groups. However, several comparative non-randomized studies were identified and assessed using the Newcastle-Ottawa Scale, demonstrating moderate to low risk of bias.

## DISCUSSION

To summarize, several key points can be highlighted. The creation of patient-specific 3D models involves multiple stages.

The process begins with acquiring high-quality medical imaging data, typically through CT (computed tomography), TEE (transoesophageal echocardiography), or MRI (magnetic resonance imaging). CT is the most frequently used modality due to its high-resolution imaging for both hard and soft tissues, its accuracy in capturing calcifications and anatomical details, and its ability to visualize surrounding cardiac structures, which is essential for precise 3D model creation. Additionally, CT’s widespread availability and relatively fast scan times make it a practical choice in clinical settings [15–19].

Images are usually stored in DICOM format, preserving all patient and scan information. Specialized software, such as 3D Slicer, Mimics, or Vesalius 3D, is used to segment the data, isolating the structures of interest. This segmentation forms the basis for creating accurate 3D models.

After segmentation, the data is converted into STL format, translating the images into a mesh of triangles that describe the model’s geometry, preparing it for 3D printing. Once STL files are prepared and necessary support structures added, printing can proceed.

Various 3D printing technologies are employed depending on the model’s purpose. According to our analysis, the most frequently used techniques were PolyJet and SLA. PolyJet offers high resolution and fine detail, producing smooth surfaces with micrometer-level precision. A key advantage is the ability to use multiple materials in a single print, creating parts with diverse properties—from rigid to flexible, transparent to opaque, or even rubber-like textures. PolyJet is particularly suited for detailed, multi-material medical models [20–22].

SLA (Stereolithography), which polymerizes liquid resin using light, is known for speed and precision, making it ideal for patient-specific cardiac models. It is often used for surgical planning of heart valves, chambers, and vasculature, allowing surgeons to assess and plan complex operations in advance [23–25].

Materials used range from commonly employed photopolymer resins to multi-material combinations. Following printing, models undergo post-processing steps, including support removal, cleaning, UV post-curing, and surface finishing, to achieve the desired appearance and mechanical properties.

The primary use is preprocedural planning, especially in complex or redo cases, where limited experience may increase replacement rates and adverse outcomes [26–28]. Models also enhance surgical training and education, improving anatomical understanding and procedural confidence among trainees and experienced surgeons alike [29, 30].

## LIMITATIONS

Several limitations should be acknowledged. From a technical perspective, the lack of standardized imaging and printing protocols remains a major challenge. In addition, currently available materials do not fully replicate the mechanical properties of biological tissue, including softness, elasticity, and tear resistance, thereby limiting the realism of the simulation. The accurate reproduction of small intracardiac structures, such as chordae tendineae and papillary muscles, remains difficult, particularly in mitral valve models with a complex subvalvular apparatus.

Practical constraints further limit clinical applicability. All 3D printing techniques are time-consuming, with production often requiring up to several days. Although cost data were not reported in the included studies, the overall expenses associated with equipment, materials, and specialized personnel are likely substantial. Moreover, there is a lack of standardized protocols for the clinical use of patient-specific models, and validation against clinical outcomes remains limited.

Methodologically, the majority of included studies demonstrated a high risk of bias due to small sample sizes and the absence of control groups, although a limited number of comparative studies showed moderate to low risk of bias. In addition, there is a lack of long-term follow-up data and a scarcity of large multicentre studies, likely reflecting the resource-intensive nature of this technology.

Important regulatory and translational challenges also persist. While 3D-printed models used for preprocedural planning or educational purposes may, in some settings, fall outside formal medical device regulatory pathways, this is accompanied by a lack of standardized quality assurance and validation frameworks. Furthermore, regulatory oversight of model production remains insufficiently defined, particularly when comparing hospital-based manufacturing with third-party commercial providers. Economic considerations represent an additional barrier, as cost coverage and reimbursement pathways are not well established. While integration into institutional training programs may allow costs to be absorbed at the hospital level, reimbursement for patient-specific models in complex clinical cases remains uncertain.

Integration into routine clinical workflows remains challenging due to time requirements, the need for specialized personnel, and the lack of standardized implementation pathways.

Taken together, these limitations highlight the early-stage nature of the field and underscore the need for standardized methodologies, robust comparative studies, and clearer regulatory and reimbursement frameworks.

## FUTURE PERSPECTIVES

Despite these limitations, 3D printing of mitral valves is highly promising. Advancements in printing techniques, materials, and software are expected to produce more precise, cost-effective, and accessible solutions.

Further research is needed to optimize multi-material printing, develop better biomimetic materials, and standardize methods. Multicentre studies and cost-effectiveness analyses will be crucial for broader clinical adoption.

Looking ahead, 3D printing could enhance patient-specific care, reduce complications, and improve surgical training. Ideally, patient-specific models could be printed before hospitalization based on pre-existing imaging data. During preprocedural planning, the surgical team could evaluate complex anatomy, confirm the diagnosis, and determine the optimal treatment strategy. The model could then be used for hands-on training, allowing the surgeon to practice the procedure and select device sizes before the operation.

Integration of 3D printing into clinical workflows is promising and is likely to become increasingly feasible as the technology develops.

## CONCLUSIONS

3D printing of patient-specific mitral valve models has emerged as a powerful tool in cardiac surgery, with applications in procedural planning, surgical training, and education. The process, which integrates advanced imaging, specialized software, and diverse printing technologies, allows for accurate replication of complex cardiac anatomy and provides valuable insights for preprocedural decision-making.

Despite its promise, several challenges remain. Technical limitations include the lack of standardized imaging and printing protocols, long production times, high costs, and the absence of biomimetic materials that fully replicate the mechanical properties of native tissues. Clinically, the field is limited by insufficient validation studies, the absence of cost-effectiveness data, and a lack of long-term or multicentre evidence.

Nevertheless, ongoing advancements in materials, multi-material printing, and software integration are expected to improve accuracy, accessibility, and cost-efficiency. With further research and standardization, 3D printing could become a routine component of mitral valve interventions, offering more precise, patient-specific care, reducing complications, and strengthening surgical training worldwide.

## Data Availability

The data that support the findings of this study are available from the corresponding author upon reasonable request.

## Abbreviations

3D TEE: three-dimensional transesophageal echocardiography
CT: computed tomography
DICOM: digital Imaging and Communications in Medicine (standard format for medical images)
DLP: digital light processing
FDM: fused deposition modeling
MR: mitral valve regurgitation
MRI: magnetic resonance imaging
PolyJet: photopolymer inkjet printing technology
SLA: stereolithography
SLM: selective laser melting
SLS: selective laser sintering
STL: standard tessellation language (file format for 3D printing)
UV: Ultraviolet
